# Validation of the de Morton Mobility Index for measuring mobility related activities in Hungarian institutionalized older adults

**DOI:** 10.1038/s41598-025-09453-6

**Published:** 2025-07-23

**Authors:** Zsigmond Gyombolai, Alexandra Zimonyi-Bakó, Anna Zsófia Kubik, Brigitta Arndt, Izabella Jónásné Sztruhár, Richárdné Mayer, András Simon, Gyöngyvér Molnár, Éva Kovács

**Affiliations:** 1 Doctoral School of Health Sciences, Semmelweis University, Budapest, Hungary; 2 Faculty of Health Sciences, Department of Morphology and Physiology, Semmelweis University, Budapest, Hungary; 3 Institute of Languages for Specific Purposes, Semmelweis University, Budapest, Hungary; 4 Faculty of Health Sciences, Department of Physiotherapy, Semmelweis University, Budapest, Hungary; 5 Old Age Home of the Municipality of Budapest, Budapest, Hungary; 6 Gizella Old Age Home, Biatorbágy, Hungary; 7 MTA-SZTE Digital Learning Technologies Research Group, Institute of Education, University of Szeged, Szeged, Hungary; 8 Faculty of Economics, Health Sciences and Social Studies, Institute of Health Sciences, Károli Gáspár University of the Reformed Church, Budapest, Hungary; 9 Faculty of Health Sciences, Semmelweis University, 17 Vas Street, Budapest, Budapest, H-1088 Hungary

**Keywords:** Mobility, DEMMI, Cross-cultural adaptation, Psychometric properties, Validity, Reliability, Dimensionality, Health care, Medical research

## Abstract

In old age, decline in mobility has a significant impact on performing activities of daily living, participating in society, and on quality of life. Mobility changes should be measured and monitored using measures with appropriate psychometric properties. The psychometrically sound de Morton Mobility Index (DEMMI) assesses a broad range of mobility related activities. The aim of this study was to develop a valid and reliable, cross-culturally adapted Hungarian version of the DEMMI scale (HU-DEMMI) among institutionalized older people. Development of the HU-DEMMI was conducted using a cross-sectional study design with an overall of 170 participants. Validity was tested via construct (established by hypothesis testing) and structural validity (explored with structural equation modeling (SEM)). Reliability was assessed using three measurement properties: internal consistency (determined by Cronbach’s alpha), test-retest reliability (by intraclass correlation coefficients (ICC)), and measurement error (standard error of measurement (SEM), minimally detectable change (MDC), limits of agreement (LoA)). Linguistic and cultural adaptation was realized successfully. As for construct validity, the HU-DEMMI showed a strong correlation with mobility related measures and a weak correlation with non-mobility measure. Significant differences in the DEMMI score were found across groups being at various levels of assistive device use. Internal consistency by Cronbach’s alpha was 0.906; the dimensionality testing revealed no violation of unidimensionality. However, a three-dimensional structure uncovered a better fit. The inter- and intra-rater ICC of DEMMI scores were 0.981 and 0.989, respectively. The SEM for inter-rater reliability test was 2.924 and the MDC_90_ was 6.803 points on the 100-point DEMMI scale. The 95% LoA showed a mean inter-rater difference of 0.2115 (− 11.252; 11.675), while a mean intra-rater difference of -1.0577 (− 9.809; 7.694). Four data points (7.69%) were identified as outliers in both cases. Maintaining institutionalized older people’s functional capacity and delaying its decline requires effective interventions. Valid and reliable determination of mobility levels is necessary to address mobility decline in a personalized way. The psychometrically robust HU-DEMMI scale developed in this study would provide this and allow for future comparisons of Hungarian research results with those of other countries and for contribution to international, multicenter research.

**Supplementary Information:**

The online version contains supplementary material available at 10.1038/s41598-025-09453-6.

## Introduction

Mobility is an essential component of both health-related quality of life and functional ability^1^. Decline in mobility has a significant impact on performing activities of daily living of older people, their participation in society and their quality of life. Mobility ability is an important indicator of the health status of patients, especially elderly patients^2,3^.

The World Health Organization (WHO) disability model, published in 2001 as the International Classification of Functioning, Disability and Health (ICF), defines mobility as the ability to change the position or location of the body^4^. Mobility includes basic positional and location changes, such as changing position in a lying position, assuming and maintaining a sitting posture, standing up from a sitting position, and maintaining a standing posture on a progressively reduced base of support. In addition, it also includes the ability to walk both short and long distances on different surfaces and to avoid obstacles^5,6^.

Those who were ambulatory prior to hospital admission spend 83% of their hospital stay in a lying position despite being ambulatory. Furthermore, more than a third of them lose their ability to perform changing body positions or locations that they previously could^7,8^.

Maintaining and improving the mobility of older people as a top priority is a shared interest for teamwork and interprofessional cooperation among all health professionals working in the field of elderly care, especially among nurses and physiotherapists^9,10^. In addition, in Hungary, the core competences of nurses and physiotherapists are also shared to some extent.

Measuring an older person’s mobility is essential for planning health care and evaluating its effectiveness. Therefore, as an indicator of functional capacity, the measurement of an older person’s mobility ability is an essential component of the geriatric assessment^11^. Mobility ability and its changes should be monitored regularly and continually using standard test methods with appropriate psychometric properties^12^.

There are several tests and scales available to measure mobility; however, one test is not sensitive enough for older people, who are also heterogeneous in terms of mobility. These tests cannot be used in higher ability groups due to ceiling effects and in lower ability groups due to floor effects^13,14^.

With its 15 progressively difficult mobility items, the de Morton Mobility Index (DEMMI) assesses a broad range of mobilities from bed and chair-mobilities, through walking ability, as well as static balance to dynamic balance^15^. DEMMI was initially developed for use in acute hospital care^15^. Further studies have also validated the DEMMI for assessing mobility in older people in other healthcare settings, such as sub-acute care, geriatric rehabilitation hospitals, and among community-living older people^16–21^. Its excellent psychometric properties have also been demonstrated in people with a variety of chronic conditions that become more common with aging, such as Parkinson’s disease, hip fractures, hip or knee osteoarthritis, and critical care patients^22–25^.

Since it was first published in English, it has been translated and validated in many other languages, including Dutch, German, Danish, Turkish, Slovenian, and Brazilian Portuguese^21,24,26–29^. However, the Hungarian version has not yet been available, and its psychometric properties have not yet been explored. A valid and reliable Hungarian version of the DEMMI scale would allow us to compare the results of research conducted in Hungary with the results of research conducted in other countries. Furthermore, the Hungarian version would also give Hungarian researchers the opportunity to participate in international, multicenter research.

The aim of this study was to develop a valid and reliable, cross-culturally adapted Hungarian version of the DEMMI scale among institutionalized older people.

Accordingly, the following research objectives (RO) were formulated.

RO1: To create the Hungarian language equivalent of the original Australian English DEMMI following the protocol of the cross-cultural adaptation process recommended by Beaton et al.^30^.

RO2: To investigate adequate validity indicators of the adapted version of the DEMMI following the quality criteria proposed by Terwee et al. and to explore whether multidimensionality can be detected^31,32^.

RO3: To assess whether the mobility of our target population of institutionalized older people can reliably be measured with the Hungarian DEMMI.

Based on our research objectives, we sought answers to seven research questions, which are detailed in the supplementary material (see Supplementary material 1, where they are marked as RQs).

## Methods

We were guided by the Strengthening the Reporting of Observational Studies in Epidemiology guidelines for observational studies and COnsensus-based Standards for the selection of health Measurement INstruments for conducting and reporting the study^33–36^.

The study protocol was approved by the Semmelweis University Regional and Institutional Committee of Science and Research Ethics (SE RKEB: 132/2023).

We conducted the study in accordance with ethical principles and the Helsinki Declaration.

All participants gave written informed consent after being informed of the aims and procedures of the study.

The adaptation process of the DEMMI was granted by the developer (Prof. Jenny Keating).

This study included two phases: (1) translation, and cultural adaptation and (2) evaluation of psychometric properties.

### Phase 1: translation and cultural adaptation

In the first phase, a Hungarian DEMMI was created following a guideline proposed by Beaton et al.^30^.

Firstly, the original English version of the DEMMI was independently translated into Hungarian by two translators, resulting in the T1 and T2 Hungarian versions. Both translators specialized in English-Hungarian medical translation. At the first consensus meeting, to produce the pre-final Hungarian version, involving Hungarian physiotherapists and nurses as well as a language specialist, the two translated scales were compared, and a preliminary Hungarian version was produced by the translators and two physiotherapists with 10–15 years of expertise in geriatric physiotherapy and one nurse with 5 years of expertise in geriatric nursing. This preliminary Hungarian version was translated back into English by two translators at a C2 proficiency level of English, a Hungarian physiotherapist and a professional Hungarian-English medical translator who were unfamiliar with the original English version of the DEMMI. At the second consensus meeting, the original DEMMI and the back-translated English versions were compared by the same experts from the first consensus meeting to produce the pre-final Hungarian version. During the pilot-testing process, we aimed to ensure that all segments of the potential users of the DEMMI were represented. Therefore, the pilot testing was carried out among a total of 33 Hungarian-speaking older adults by health and social care professionals working in geriatric care including a physiotherapist with 15 years of experience in geriatric physiotherapy working in homecare, a nurse with 20 years of experience in geriatric nursing working at an acute hospital setting, a physiotherapist with 5 years of experience in geriatric physiotherapy as well as a nurse with 5 years of experience in geriatric nursing working at a chronic hospital setting, and a caregiver with 10 years of experience in geriatric care working at a long-term care setting. Professionals were asked to rate the comprehensibility of the Tasks, Scoring, Item instructions, and the Protocol for administration sections of the DEMMI on a 5-point scale, where a score of 1 meant not at all and a score of 5 meant completely understandable. They were also asked to explain why they found a section of the DEMMI problematic and to suggest ways to better understand it.

In addition, a think-aloud method was used to assess whether the tasks, item instructions and Protocol for administration of the DEMMI were understood as intended^37^. During the think-aloud, a nurse, a physiotherapist, and a caregiver were asked to read the tasks, item instruction, protocol for administration aloud and verbalize their thoughts while responding to each item. After their informed consent, the professionals’ think-aloud responses were video-recorded. Any uncertain points indicated during pilot-testing were corrected in the final Hungarian version of DEMMI.

### Phase 2: evaluation of psychometric properties

#### Participants

Our cross-sectional study was conducted between June 2023 and June 2024 on a convenience sample of older adults in three long-term care institutions located in Budapest, Hungary.

Out of a total of 732 residents of a state-run institution in the capital, a church-run institution in the capital, and a church-run institution in a small town near the capital, we included residents aged 60 and over who had lived in the institution for at least 4 weeks. The exclusion criteria were as follows: sensory aphasia, contraindication to mobilization in the medical records, sensory or cognitive impairment in communication that made it impossible to understand the instructions and perform the tasks in the test (i.e., severe dementia), anticipated death in the immediate future (based on physician’s judgment), and refusal to participate.

From a total of 457 eligible residents of the three institutions, study participants were randomly selected as follows. Each eligible resident was paired with a list of random numbers between 1 and 457, generated by a computer. The random numbers were then sorted in ascending order and the first 170 participants were recruited into the study.

With the aforementioned randomization method, 55 participants were selected from the total sample, who were reassessed to examine inter-rater and intra-rater reliability. Two physiotherapists (Assessors A and B) with five and seven years of experience in geriatric physiotherapy respectively, assessed the randomly selected participants separately. Assessor A performed the first DEMMI assessment and Assessor B performed the second assessment on the participants two hours later. After seven days, Assessor A performed the DEMMI again on the same participants.

#### Measurements

Participants’ sociodemographic data, including age and sex, length of stay in the institution and health-related data, including weight, height, Mini Mental Examination, and chronic diseases, were extracted from care records.

In addition to the implementation of DEMMI, functional mobility, functional lower limb strength, the amount of personal assistance needed for ambulation, as well as physical disability in activities of daily living (ADL), and fear of falling were assessed.

To assess functional mobility, the Timed Up and Go (TUG) test was used. This test measures the time (in seconds) necessary for the participant to stand up from a standard armchair (approximate seat height 46 cm; arm height 65 cm), walk to a cone 3 m from the chair, turn back, walk back to the chair, and sit down again. The participants were instructed to ‘walk at a comfortable and secure pace’. They were allowed to wear their usual footwear, use their usual walking aids, and support themselves on the arm of a chair to stand up, but no physical assistance was delivered for them. After a trial to get familiar with the test, two consecutive performances were timed, and their mean was recorded. Between tests, they were allowed to rest for 30 s if required^38^.

To assess functional lower limb strength, the 30 s Sit to Stand Test was used. This test counts the number of standard sit-to-stand in 30 s. The participants were in a seated position on an armless chair with its back placed against the wall. Their arms were folded across the chest. They were asked to stand up and sit down as many times as possible within 30 s without using the upper limb. The standard standing up meant that the trunk was vertical and knees were extended. One trial before measurement was allowed to practice the correct execution^39^.

To assess the amount of personal assistance needed for ambulation, the Functional Ambulation Category was used. This test scores on a 6-point scale how much human personal assistance the patients need during walking, whether they use a walking aid or not. The higher the score, the more independent the participant^40^.

To assess physical disability in ADL, the Barthel Index was used. This scale measures the degree of assistance needed by a participant for ten basic activities of daily living (ADL) including feeding, bathing, grooming, dressing, bowel, bladder, toilet use, transfers bed-to-chair-and-back, mobility on level surfaces, and stair negotiation. The higher the score, the more independent the participant. The administration was performed by interviewing the participants and/or their caregivers^41,42^.

To assess fear of falling, the short FES-I was used. With its seven items, it measures the level of concern for falling during both social and physical activities in and outside the home. The total score can vary from 7 to 28 points with a higher score indicating a higher level of concern for falling^43^.

The DEMMI is a performance-based scale including 15 items that can be divided into 5 groups, comprising mobility activities in bed (3 items), chair-based mobilities (3 items), static balance activities (4 items), walking/ambulation (2 items) and dynamic balance activities (3 items). Participants’ mobility abilities are assessed using either a 2-point or 3-point scale. For items 1, 2, 4, 6–10, and 13–15, scores are assigned on a dichotomous (2-point) scale, with values of 0 or 1, depending on whether participants can perform the task independently or not. For items 3, 5, 11, and 12, scores are assigned on an ordinal 3-point scale, with values of 0, 1, or 2, depending on whether participants can complete the task independently, with assistance only, or not at all. The aggregate scores result in raw scores ranging from 0 to 19 points. On the DEMMI form, a conversion table is provided for the transformation of the raw score into a total interval DEMMI score, which ranges from 0 to 100 points, with higher scores indicating a higher level of mobility^15^. Although formal training is not required for its administration, the DEMMI education resources available on the website include instructional videos, example applications of the DEMMI in four different healthcare settings, and an instructional handbook^44^.

#### Statistical analyses

Data are presented as numbers and percentages for discrete data and as means and standard deviations (SD) or median and interquartile range (IQR) for continuous data, as appropriate to the distribution. The distribution of our data was checked using the Kolmogorov-Smirnov test.

The psychometric properties (validity and reliability) of the HU-DEMMI were assessed following the quality criteria published by Terwee et al.^31^ and using the COSMIN recommendations^34–36^.

To judge the floor and ceiling effects, the number of participants with the highest and lowest possible scores was identified. The floor and ceiling effects were considered to be present if at least 15% of the participants reached the minimum or maximum score, respectively^31^.

## Reliability

The reliability of the HU-DEMMI was assessed using three measurement properties: internal consistency, test-retest reliability, and measurement error.

Internal consisitency: To assess internal consistency, Cronbach’s alpha value was calculated. Values between 0.7 and 0.95 were considered acceptable^31^.

Test-retest reliability: To investigate test-retest reliability, the inter-rater reliability indicator was determined by calculating the intra-class correlation coefficient (ICC) in a two-way random effect ANOVA model. A total of 55 older adults were selected to ensure a minimum sample size of 50. This sample size was chosen to achieve the expected ICC of 0.70 with a power of 0.80 and a significance level of 0.05, while also accounting for a 10% dropout rate^31^.

Measurement error: To determine measurement error, the standard error of measurement (SEM) of repeated measures was first calculated using the formula SEM = SD*√(1-ICC). This SEM was then used to calculate the minimally detectable change at the 90% confidence level (MDC90) using the formula 2.363 × SEM. Furthermore, Bland-Altman plots, which display the difference between the HU-DEMMI scores and the means of the corresponding HU-DEMMI scores, were employed to visually demonstrate the level of agreement between raters and test-retest measures. The 95% limit of agreement was estimated as ± 1.96 × SD of the mean difference scores^45^.

Construct validity of the HU-DEMMI was established on the basis of hypothesis testing strategy with following hypotheses.

H1: A strong positive correlation (rho ≥ 0.70) is expected between the HU-DEMMI scores and Barthel Index values, as published by others^15,19,46^.

H2: A strong negative correlation (rho ≥ 0.70) is expected between the HU-DEMMI score and the TUG test result. This hypothesis is based on evidence from Braun et al., and Jans et al., who demonstrated strong negative correlations between these measures^24,46,47^.

H3: A strong positive correlation (rho ≥ 0.7) is expected between the HU-DEMMI score and 30 s Sit-to-Stand Test. Jans et al. reported a correlation of rho = −0.69 between the DEMMI scores and the Chair Rise Time in older patients with knee or hip osteoarthritis^24^.

H4: A strong positive correlation (≥ 0.70) is expected between the HU-DEMMI score and FAC score. Our hypothesis was based on previous studies of Braun et al., who reported a correlation ranging from rho = 0.7 to 0.93 in various older populations^21,46–48^.

H5: A moderate negative correlation (0.4 ≤ rho < 0.7) is expected between the HU-DEMMI score and the FES-I score as Braun et al. reported a correlation of rho = −0.68 between the DEMMI and the FES-I scores in patients admitted to a sub-acute inpatient geriatric rehabilitation hospital^21^.

H6: A weak negative correlation (≤ 0.3) is expected between the HU-DEMMI score and Charlson Comorbidity Index. This hypothesis was based on previous studies which consistently found correlations below 0.3 between the DEMMI scores and comorbidity^19,21,22^.

H7: The HU-DEMMI score would be higher in people who are able to walk independently compared to those using a walking cane, frame or rollator as well as those who are non-ambulatory. Braun et al. found a significant difference in DEMMI score between those who were able to ambulate independently in the hospital and those requiring physical support, supervision, or who were non-ambulatory^21^.

The correlations were analyzed using Spearman correlation coefficients categorized as weak (≤ 0.39), moderate (0.40–0.69), or strong (≥ 0.70)^49^.

Kruskal-Wallis ANOVA test was used to determine the difference in the HU-DEMMI scores between (1) older adults who were able to walk independently of any walking aids; (2) who were able to walk with a walking stick/cane; (3) who were able to walk either with frame or rollator, and (4) who were not able to walk at all. In the case of a significant result, multiple pairwise comparisons were made using Mann-Whitney U tests with Bonferroni-corrected p-value. During multiple comparisons, the alpha level was adjusted with a Bonferroni correction and the p-value for significance was set to 0.008.

Construct validity is considered good if more than 75% of the pre-defined hypotheses are confirmed.

Structural validity was established by means of structural equation modeling. This procedure monitors how well the data confirmed the theory and reflected the structure of the construct under investigation, and to investigate the dimensionality of the DEMMI, that is, how well the data supported the concept of the theoretical model behind the instrument. χ^2^ values, an absolute fit index (the root mean square error of approximation, RMSEA), and two incremental fit indices (the Tucker–Lewis Index, TLI, and the comparative fit index, CFI) were computed to evaluate model fit. We used the preferred estimator for categorical variables, Weighted Least Squares Mean and Variance (WLSMV) adjusted^50^. According to Hu and Bentler, a CFI and a TLI value above 0.95 and a RMSEA below 0.06 indicate a good global model fit^51^. In order to directly compare the possible theoretical models, we carried out a special Chi2-difference test in Mplus^50^.

A partial credit model was used for scaling the data and identifying a hierarchy of mobility items ranked from the easiest to the hardest. This model is an extension to Rasch’s logistic model and is suitable for analyzing items scored polytomously not having the same step parameters^52^. Differential Item Functioning (DIF) is a form of item bias that exists when individuals with the same ability perform differently on an item supposing the effect of another variable. DIF for the DEMMI was investigated for age (< 80 years and 80 + years), gender, and CCI score (0–6 and 7+).

Since SEM relies on the CFA method, the appropriate sample size was set at 10 subjects per item. Considering a 10% dropout rate, a total of 170 participants were required. Statistical analyses were performed with SPSS 18.0 software, except for structural equation modeling and partial credit model analyses, which were conducted with MPlus and ConQuest.

P-values less than 0.05 were accepted as significant.

## Results

*Results for research question 1 (RQ1): Which equivalent terms are required to be chosen during the process of linguistic adaptation to ensure cross-cultural validity?*

### Forward translation and the preliminary version

During the forward translation stage, some minor linguistic differences have arisen.

Regarding the term ‘bridge’ in item 1, since the movement of ‘bridge’ in Hungarian is used to mean the lifting of hips or bottom, ‘medenceemelés’ (literally ‘pelvis elevation’) was used instead of ‘híd’ (literally ‘bridge’).

The term ‘tandem stand’ in item 10 was not clear to the experts who were not physiotherapists. For this reason, an additional explanatory term (‘tyúklépésben állás’ literally ‘hen stepping’) was inserted after ‘tandem stand’.

Regarding ITEM INSTRUCTION 4, the person’s sitting position was not clear, whether or not it is allowed to lean against the backrest. After observing the demo video about performing DEMMI in the educational resources (https://www.demmi.org.au/resources/), the following additional explanatory sentence was added: ‘The back and arms must not come into contact with the chair’.

### Pilot testing

The mean age of the pilot test participants was 80.18 years (SD 7.88, range 65–92), 70% of whom were female (*n* = 23). There were 2 participants with myocardial infarct, 7 with high blood pressure, 9 with hemiplegia, 17 with chronic pulmonary disease, 2 with rheumatological disease, 10 with gastrointestinal disease, 12 with renal disease, 11 with diabetes, and 11 with mild to moderate cognitive disease.

During the pilot testing, it was observed that when reading the first page of the DEMMI sheet, some respondents only understood the short instructions for the tasks after their attention was drawn to the ITEM INSTRUCTIONS on the other side of the sheet. To solve this problem, after the title of the sheet (de Morton Mobility Index DEMMI), the following sentence was inserted: ‘Please read carefully the information on page 2!’ The same problem was detected regarding the instructions. To solve this problem, the ‘Definition’ section at the bottom of the page was moved after the title of ITEM INSTRUCTION. Furthermore, regarding the PROTOCOL FOR ADMINISTRATION OF THE DEMMI, to facilitate the interpretation of the text, key phrases in sentences have been highlighted in bold font. No additional changes were made to the Hungarian version of DEMMI.

The version developed on the consensus meeting after the pilot phase was named HU-DEMMI. After the developer of DEMMI (Prof. Jenny Keaton) agreed with the modifications in the instrument, the final version was confirmed and named HU-DEMMI. (see Supplementary material 2).

*Results for research questions 2a and 2b (RQ2a and RQ2b): Does the HU-DEMMI show acceptable construct validity*,* based on an association between the DEMMI and another mobility-related measure? Does the HU-DEMMI show acceptable construct validity*,* based on its correlation with non-mobility measures?*

Of the 170 participants recruited, 3 were hospitalized, 6 were eventually not motivated to participate and 3 died, making a total of 158 participants’ data available for analysis in this study. Their characteristics are presented in Table 1.

**Table 1:** Demographic and clinical characteristics of the participants.

Characteristics	Values	No. of participants
Age (years) *M* ± *SD*	84.14 ± 8.93	158
Female, n (%)	135 (85.4%)	158
LOS (month) *M* ± *SD; Mdn* (*Q*_*1*_; *Q*_*3*_)	42 ± 33.88; 32 (15; 60)	158
BMI (kg/m^2^) *M* ± *SD*	25.49 ± 5.65	146
CCI (scores) *M ± SD; Mdn (Q*_*1*_; *Q*_*3*_*)*	5.93 ± 1.69; 6 (5; 7)	158
BI (scores) *M ± SD; Mdn (Q*_*1*_; *Q*_*3*_*)*	72.84 ± 27.35; 80 (60; 90)	158
MMSE (scores) *M ± SD*	21.89 ± 5.41	150
TUG (sec) *M ± SD; Mdn (Q*_*1*_; *Q*_*3*_*)*	29.50 ± 20.61; 35 (16.5; 35.25)	130
FAC (scores) *M ± SD; Mdn (Q*_*1*_; *Q*_*3*_*)*	3.26 ± 1.78; 4 (2; 5)	158
30sSTSTest (sec) *M ± SD; Mdn (Q*_*1*_; *Q*_*3*_*)*	7.13 ± 3.35; 7 (4; 9)	82
DEMMI (scores) *M ± SD; Mdn (Q*_*1*_; *Q*_*3*_*)*	49.43 ± 20.27; 52.50 (39; 62)	158
Short FES-I (scores) *M ± SD; Mdn (Q*_*1*_; *Q*_*3*_*)*	13.54 ± 5.77; 12 (8; 18)	158

LOS, Length of Stay (in the institution); BMI, body mass index; CCI, Charlson Comorbidity Index; BI, Barthel Index; MMSE, Mini-Mental State Examination; TUG, Timed Up and GO; FAC, Functional Ambulation Category; 30sSTST, 30 s Sit to Stand Test; DEMMI, de Morton Mobility Index; FES-I, Falls Efficacy Scale - International; M, mean; SD, Standard Deviation; Mdn, Median Note: Q1, lower quartile; Q3, upper quartile.

Of the 132 ambulatory participants, 25 (15.8%) used a walking cane, and 69 (43.7%) used a walker (frame or rollator). Thirty-eight participants (24.1%) were able to walk without a walking aid and 26 (16.5%) participants were non-ambulatory.

The mean score on the DEMMI was 68.93 ± 14.3.

Table 2 shows Spearman’s *rho* correlation coefficients between DEMMI scores and measures of related or distinct constructs. The DEMMI showed a strong positive correlation with the Barthel Index, 30 s Sit to Stand Test, as well as Functional Ambulatory Category, and a strong negative correlation with the TUG test and short FES-I. The DEMMI showed a weak negative correlation with the Charlson Comorbidity Index.

**Table 2:** Correlations between DEMMI scores and measures of related or distinct constructs.

No. Hypothesis	Measures	No. of participants	Spearman’s rho	95% CI	*p*-value	Confirmed
H1	Barthel Index	158	0.764	0.69, 0.822	< 0.0001	Yes
H2	TUG	130	- 0.711	−0.786, −0.614	< 0.0001	Yes
H3	30sSTST	82	0.715	0.589, 0.806	< 0.0001	Yes
H4	FAC	158	0.850	0.8, 0.888	< 0.0001	Yes
H5	Short FES-I	158	- 0.504	0.378, 0.612	< 0.0001	Yes
H6	CCI	158	− 0.232	−0.37, −0.079	0.003	Yes

BI, Barthel Index; TUG, Timed Up and Go test; 30sSTST, 30 Second Sit to Stand Test; FAC, Functional Ambulation Category; FES-I, Falls Efficacy Scale – International; CCI, Charlson Comorbidity Index; CI, Confidence Interval.

Results for research question 2c (RQ2c): *Does the HU-DEMMI show acceptable construct validity*,* based on differences in known-groups*?

**Fig. 1:**
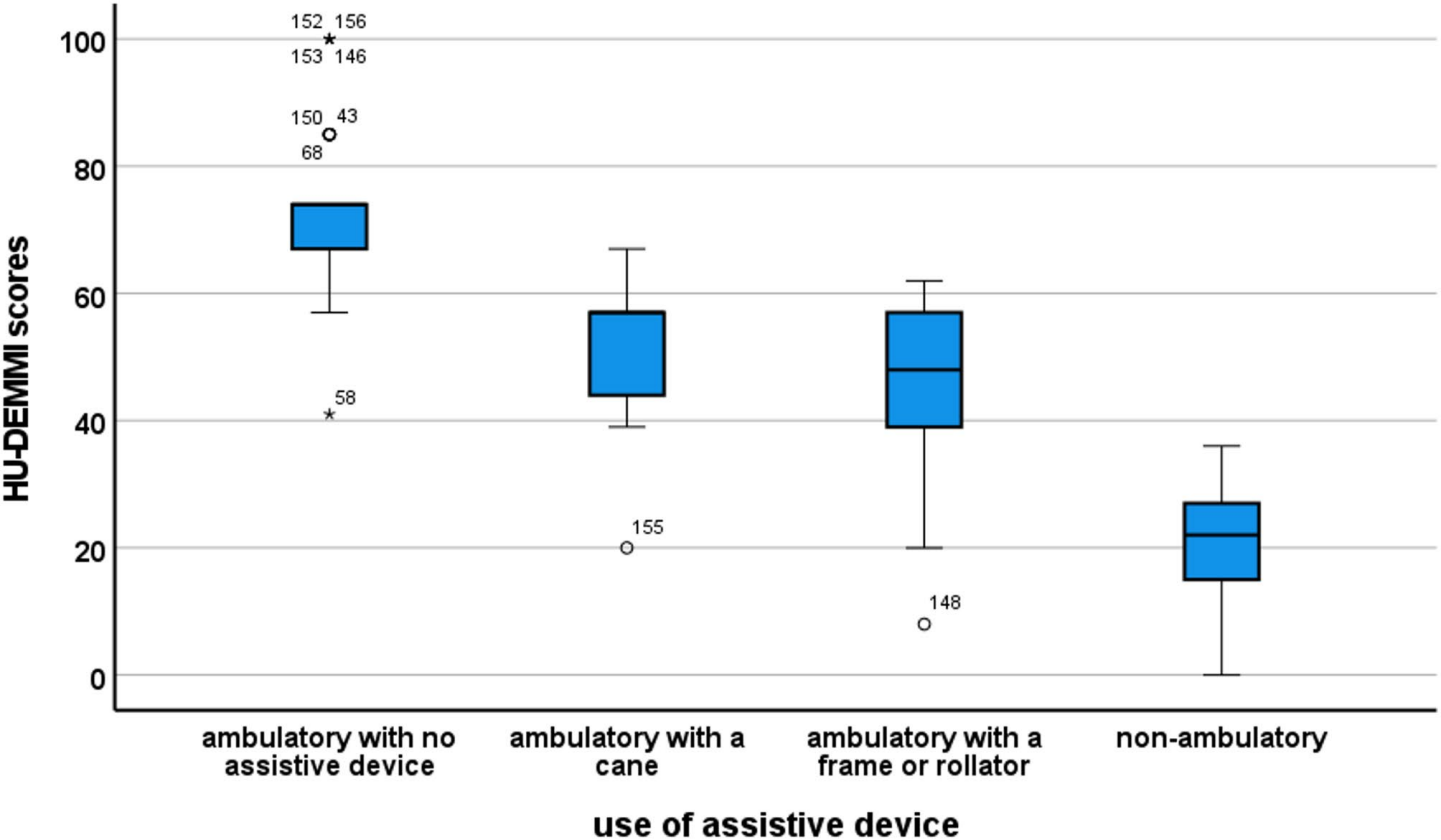
Boxplot diagrams displaying groups formed by the use of assistive device.

A Kruskal-Wallis test indicated that there was a significant difference in HU-DEMMI scores across the four groups formed based on the use of walking aid showing different mobility levels, *χ*^*2*^ (3, *N* = 158) = 112.89, *p* < 0.001. The median HU-DEMMI scores were 74 for those who could walk independently, 57 points for those who could walk with a cane, 48 points for those who could walk with a frame or rollator, and 22 points for those who could not walk. Figure 1 displays the box-plots for the comparison of the four groups of different mobility abilities.

Multiple pairwise comparisons by Mann-Whitney *U* tests were done with the following results by level of mobility:

1. Groups with the ability to walk independently *versus* ambulatory with a walking cane.

Based on the result of the *U* test, the mobility measured with the HU-DEMMI can significantly be decreased (*Mdn* = 57) if one needed the use of a walking cane compared to those with the ability to walk independently (*Mdn* = 74) with a large effect size (Mann-Whitney *U* = 49 *Z* = −6.07 *p* < 0.0005 (1-tailed) *r* = 0.804).

2. Groups with the ability to walk independently *versus* ambulatory with a walker (frame or rollator).

Similarly, as expected, when having compared the mobility of those being able to walk independently (*Mdn* = 74) and of those who needed a frame or rollator (*Mdn* = 48), a statistically significant and large decrease was verified (Mann-Whitney *U* = 77 *Z* = −8.070 *p* < 0.0005 (1-tailed) *r* = 0.78).

3. Groups with the ability to walk independently *versus* non-ambulatory group.

Not surprisingly, the greatest difference in mobility levels measured with HU-DEMMI was detected between those who walked independently (*Mdn* = 74) and those in the non-ambulatory group (*Mdn* = 22), with a strong effect on mobility when one could not walk (Mann-Whitney *U* = 0.00 *Z* = −6.81 *p* < 0 0.0005 (1-tailed) *r* = 0.851).

4. Groups ambulatory with walking cane *versus* ambulatory with a walker (frame or rollator).

On the other hand, there was no statistically verifiable difference in the HU-DEMMI scores between those using a walking cane (*Mdn* = 57) and those using a frame or a rollator (*Mdn* = 48) (Mann-Whitney *U* = 596 *Z* = −2.309 *p* = 0.021 (2-tailed) *r* = 0.238). (The p-value of *U*-statistics is 0.0105 (1-tailed), cf. Bonferroni-corrected alpha (α_b_) = 0.004 (1-tailed)).

5. Group ambulatory with walking cane *versus* non-ambulatory group.

Similarly to pairwise comparison #3, the people who could walk with a cane performed better in the HU-DEMMI (*Mdn* = 57) than non-ambulatory participants (*Mdn* = 22). The difference could be verified statistically as well, with a strong effect on the decline of HU-DEMMI scores of non-ambulatory people (Mann-Whitney *U* = 15.00 *Z* = −5.882 *p* < 0.0005 (1-tailed) *r* = 0.824).

6. Group ambulatory with a walker (frame or rollator) *versus* non-ambulatory group.

Our last pairwise comparison gives a similar result to our comparison in points #3 and #5. We compared the median HU-DEMMI score (*Mdn* = 48) of people using a frame or a rollator with the median HU-DEMMI score (*Mdn* = 22) of people who were not ambulatory, as there was a strong statistically proven effect of reduced mobility (Mann-Whitney *U* = 45.00 *Z* = −7.134 *p* < 0.0005 (1-tailed) *r* = 0.732).

All pre-specified hypotheses (100%) regarding the correlations between the HU-DEMMI and various functional measures, including both mobility-related and non-mobility-related ones, as well as known group differences, were fully confirmed.

Results for research questions 2 d and 2e (RQ2d and RQ2e): *How well does the original unidimensional DEMMI fit our observed data? Is the requirement of unidimensionality met or somewhat compromised*,* or if multidimensionality is likely*,* does a three-dimensional or a five-dimensional model fit the data better than the unidimensional model?*

## Dimensionality

The 1-dimensional model with all indicators combined under one general factor showed a good fit compared to the cut-off values recommended by Hu and Bentler^51^. A special χ^2^-difference test (Chi-Square Test for Difference Testing) in Mplus indicated that the 3-dimensional model (dim 1: items 1–6; dim 2: items 7–10; dim 3: items 11–15), as well as the 5-dimensional model, fitted significantly better on a *p* < 0.01 level and on a *p* < 0.001 level than the 1-dimensional model, but the 3-dimensional model and the 5-dimensional model did not differ significantly (1 dim vs. 5 dim: Chi-Square Test for Difference Testing = 25.226, *df* = 10, *p* = 0.0049; 1 dim vs. 3 dim: Chi-Square Test for Difference Testing = 18.788, *df* = 3, *p* = 0.0003; 3 dim vs. 5 dim: Chi-Square Test for Difference Testing = 4.712, *df* = 7, *p* = 0.6951). The goodness of fit indices for testing dimensionality of DEMMI are presented in Table 3.

**Table 3:** Goodness of fit indices for testing dimensionality of DEMMI.

Model	χ^2^	df	*p*	CFI	TLI	RMSEA	CI (RMSEA)
1-dimensional	153.819	90	0.001	0.987	0.985	0.067	0.048–0.085
3-dimensional	139.009	87	0.001	0.990	0.988	0.062	0.042–0.080
5-dimensional	138.345	80	0.001	0.989	0.985	0.068	0.048–0.087

*df*, degrees of freedom; CFI, Comparative Fit Index; TLI, Tucker Lewis Index; RMSEA, Root Mean Square Error of Approximation; *Note*: *χ2* and *df* are estimated by WLSMV adjusted.

Within the context of Rasch modeling, an item is deemed to exhibit differential item functioning (DIF) if the response probabilities for that item cannot be fully explained by the ability of the tested person and a fixed set of difficulty parameters for that item. For testing DIF, the polytomous items test and the partial credit model were used.

The interaction tasks*gender models the variation in the difficulty of the task between the two genders; and the gender*tasks*step models differing step structures for both task and gender.

The task*gender interaction was not significant, so this test did not exhibit DIF in the overall item difficulty (*χ*^*2*^ test for equality of parameters = 9.886, *df* = 14, *p* = 0.770). Having investigated the task*CCI interaction, item 10 had to be excluded as there was no data for CCI code 1. By analyzing the remaining 14 items, the task*CCI interaction was not found significant, so this test did not exhibit DIF in the overall item difficulty (*χ*^*2*^ test for equality of parameters = 11.809, *df* = 13, *p* = 0.543). Three items (items 1, 9, and 10) showed significant DIF by age. Excluding these items, task*age interaction was not significant, so this test did not exhibit DIF in the overall item difficulty (*χ*^*2*^ test for equality of parameters = 17.016, *df* = 11, *p* = 0.107).

Based on estimated item difficulties, item 2 (roll) was the easiest, while item 10 (tandem stand with eyes closed) was the most difficult task (−6,64 logit and 9.997 logit respectively).

A high positive logit location (e.g. standing on toes) indicates harder item difficulty compared to a negative logit location (e.g. bridging). Table 4 presents the item difficulty index and fit statistics.

**Table 4:** Item difficulty index and fit statistics.

VARIABLES			WEIGHTED FIT		
Item	ESTIMATE	ERROR ^a^	MNSQ ^b^	CI	T ^c^
1. Bridge	−4.904	0.304	1.59	(0.54, 1.46)	2.2
2. Roll onto side	−6.640	0.326	0.63	(0.45, 1.55)	−1.4
3. Lying to sitting	−5.124	0.248	1.14	(0.54, 1.46)	0.7
4. Sit unsupported	−5.547	0.313	1.21	(0.54, 1.46)	0.9
5. Sit to stand from chair	−2.674	0.203	0.98	(0.69, 1.31)	−0.1
6. Sit to stand without using arm	0.711	0.218	1.17	(0.71, 1.29)	1.1
7. Stand unsupported	−2.675	0.245	0.89	(0.64, 1.36)	−0.6
8. Stand feet together	1.330	0.222	1.19	(0.71, 1.29)	1.3
9. Stand on toes	8.599	0.320	1.67	(0.53, 1.47)	2.4
10. Tandem stand with eyes closed	9.997	0.363	1.85	(0.19, 1.81)	1.8
11. Walking distance	−2.414	0.187	1.05	(0.62, 1.38)	0.3
12. Walking independence	1.271	0.201	1.20	(0.74, 1.26)	1.4
13. Pick up pen from floor	0.668	0.220	1.07	(0.71, 1.29)	0.5
14. Walk 4 steps backwards	0.731	0.220	0.94	(0.71, 1.29)	−0.4
15. Jump	6.671*	0.981	1.57	(0.57, 1.43)	2.3

An asterisk next to a parameter estimate indicates that it is constrained; Separation Reliability = 0.997; *χ*^*2*^ test for equality of parameters = 3462.212, *df* = 14, *p* = 0.000; ^a^Quick standard errors have been used; ^b^MNSQ: mean square fit values; ^c^Fit T statistics.

Results for research question 3 (RQ3): *Is the Hungarian version of the DEMMI applicable and reliable for measuring mobility among older people living in long-term care facilities with different levels of mobility ability?*

There were no floor or ceiling effects because only four participants (2.5%) were given the highest total score of 100, and four of the participants (2.5%) were given the lowest, null score.

The Cronbach’s alpha coefficient for the HU-DEMMI was 0.906 (95% CI: 0.883–0.926), indicating acceptable internal consistency.

Test-retest reliability: Of the 55 people originally selected, 52 participants took part in all three assessments. The ICC model 2.1 for inter-rater reliability of DEMMI scores with a 95% confidence interval was 0.981 (0.966–0.989). The ICC model 2.1 for intra-rater reliability of DEMMI scores with a 95% confidence interval was 0.989 (0.980–0.993).

**Fig. 2:**
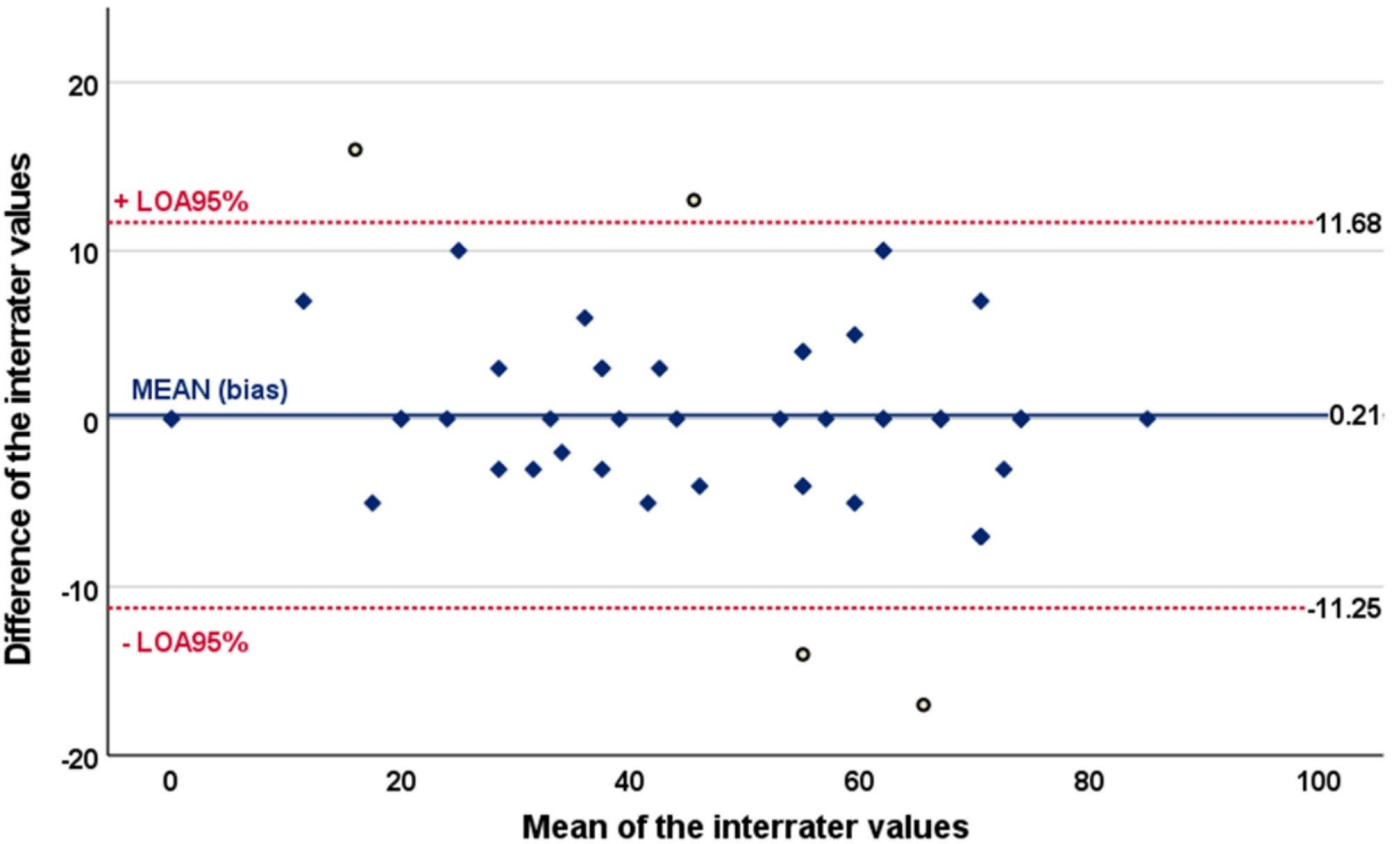
Bland-Altman plot for interrater reliability testing of HU-DEMMI. *Legend* Bland–Altman plot showing differences of measurements against their means for inter-rater reliability. The solid (blue) line represents the mean difference (bias) between the two scores, and the dotted (red) lines represent the 95% limits of agreement (LOA95%).

**Fig. 3:**
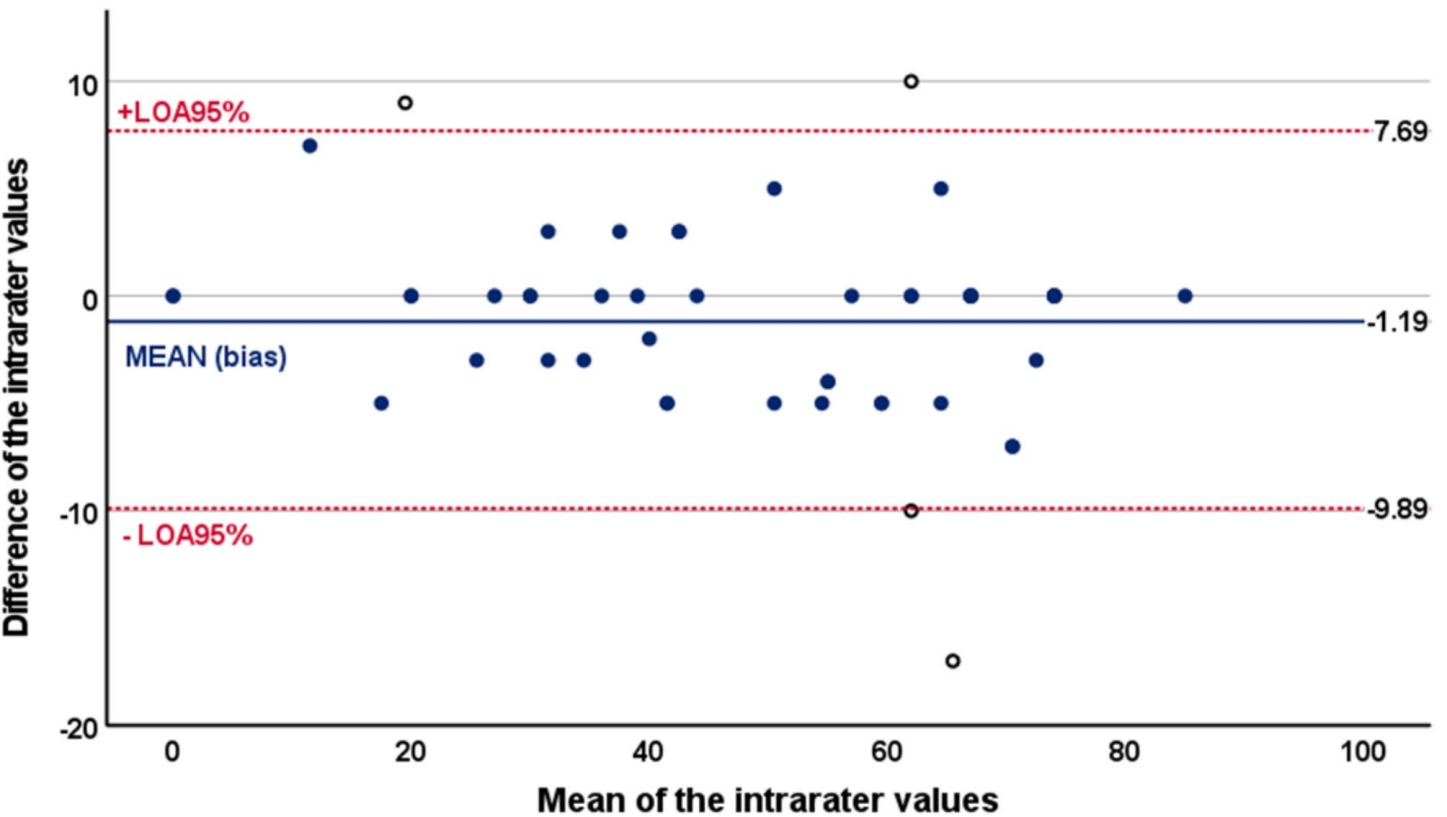
Bland-Altman plot for intrarater reliability testing of HU-DEMMI. *Legend* Bland–Altman plot showing differences of measurements against their means for intra-rater reliability. The solid (blue) line represents the mean difference (bias) between the two scores, and the dotted (red) lines represent the 95% limits of agreement (LOA95%).

Measurement error: Using a pooled SD of 21.21, the SEM for the inter-rater reliability test was 2.924 and the MDC_90_ with a 95% confidence interval was 6.803 (0.897–12.709) points on the 100-point DEMMI scale. This means that a change of at least 7 points in a patient’s DEMMI score is required for the clinician to be 90% sure that there has been a real change in the patient’s condition. The Bland–Altman plots of the inter- and intra-rater reliabilities are presented in Figs. 2 and 3. The mean inter-rater difference was 0.2115, with 95% limits of agreement ranging from − 11.252 to 11.675. Four outliers (7.69%) were identified. The mean intra-rater difference was − 1.0577, with 95% limits of agreement ranging from − 9.809 to 7.694. Similarly, four data points (7.69%) fell outside this range.

## Discussion

The aim of this cross-cultural validation study was to develop the Hungarian version of the DEMMI for older adults living in an institution providing long-term care and to examine its psychometric properties.

There were only minor comprehension problems during the cross-cultural adaptation process.

Some terms in the items were slightly modified to ensure that Hungarian people understood the task accurately and performed it correctly. Only one explanatory sentence was added to the instructions to clarify the exact meaning of the task in the fourth item. No other changes have been made apart from this.

So far, six studies have examined the psychometric properties of various national versions of DEMMI used in different clinical settings^15,21,24,27–29^.

The original DEMMI’s psychometrics were evaluated in Australian older people (mean age 81.2 years) admitted to an inpatient rehabilitation facility^15^. In terms of age, the Dutch, German, and Hungarian samples were the most similar to this original sample (75, 80 and 83 years respectively)^21,24^. The Dutch DEMMI was validated among older people awaiting total hip or knee arthroplasty while the German DEMMI among patients admitted to a sub-acute inpatient geriatric rehabilitation hospital^24^. However, our sample consisted of older adults living in institutions providing long-term care. The older adults admitted to these facilities were not admitted for the same indication or reason; consequently, significant differences in their mobility ability can partly be explained by this. The mean age of the Brazilian sample was more than 10 years lower than that of our sample^29^. They were hospitalized for various clinical reasons such as pulmonary and cardiovascular diseases or neoplasia. The Slovenian sample consisted of people aged between 18 and 85, who were admitted to inpatient rehabilitation due to musculoskeletal impairments^27^.

Indicators from studies on different samples and in different clinical settings show a high degree of similarity; it should be noted, however, that the results from these samples are only comparable to a limited extent.

The construct validity of the HU-DEMMI was confirmed as a strong correlation was proved with other mobility-related measures (Barthel index, Sit-to-stand, FAC, TUG and FES-I). These results are similar to those found in the studies conducted by de Morton et al., Jans et al., Braun et al., Zupanc, and Yürük et al.^15,21,24,27,28,46–48^.

Furthermore, it showed a weak correlation with the measures (CCI) not related to mobility. These results were found in the studies carried out by de Morton et al. and Braun et al.^15,21^.

As for the differences of the DEMMI scores amongst patient subgroups according to their use of walking aids our results are in line with the results of the studies previously conducted among older people admitted either to a sub-acute inpatient geriatric rehabilitation hospital or to an acute care setting^21,27^. After examining the multiple pairwise post-hoc comparisons, we found that mobility levels in our sample are well separated when categorized as (1) older people who can walk without walking aids; (2) older people who can walk with assistive devices (cane, rollator, or frame) and (3) older people who cannot walk. When comparing the mobility level measured by HU-DEMMI with these categorized groups, our predefined hypothesis is confirmed. However, according to the findings of the present study we can reach erroneous conclusions if we automatically consider the use of different assistive devices as a direct representation of different mobility levels. The preference for the choice of walking aids may be influenced by a number of factors. Only part of this preference is reflected in the functional ability of the older person.

Our results support previously published findings that the DEMMI scale is a unidimensional construct as the data fit the model well. In practice, however, it has been found that unidimensionality can be compromised for a number of health outcome scales (e.g., the Functional Independence Measure) that, although originally designed to measure a single variable, are composed of subsets of items that measure slightly different aspects of that variable^32^. The original DEMMI was designed to measure mobility as a single performance variable and has been shown to meet the requirement of unidimensionality, an indicator of the internal consistency of the construct. Yet, its items cover a wide range of mobility activities requiring different levels of mobility abilities, and the DEMMI can therefore be considered as a not fully homogeneous scale. Due to the subsets of items, multidimensionality may be detected when applied to a sample similar to ours. Testing a 3-dimensional (dimension 1: bed- and chair-mobilities; dimension 2: static balance activities; dimension 3: walking and dynamic balance activities) and a 5-dimensional model (dimension 1: bed-activities; dimension 2: chair-activities; dimension 3: static balance activities; dimension 4: walking; dimension 5: dynamic balance activities), significant improvements were observed in the fit indices. Since the 3-dimensional and 5-dimensional models were not statistically different, the simpler 3-dimensional model was accepted as the best fit to our data.

In other words, our results confirm that the unidimensional model fits data from a population of older people living in long-term care as well as data from a population of acute, sub-acute patients or from older people living at home. Simultaneously, the indicators from a three-dimensional model derived from further analysis of our sample from the long-term care population appear to be even better than those from the unidimensional model.

Just as in the Australian sample, the task of item 10 was the most difficult one (tandem standing with eyes closed) in the Dutch, German, and Hungarian samples^15,21,24^.

In the Slovenian sample, however, item 15 (jump) proved to be the most difficult one^27^.

The easiest task in the Australian, Dutch, and Slovenian samples was of item 4 (sitting unsupported); in the German sample the task of item 3 (lie to sit), while in the Hungarian sample, the task of item 2 (roll) was the easiest one^15,21,24,27^.

In both the Australian and the Hungarian samples, the three most difficult tasks were of item 15 (jump), item 9 (stand on toes), and item 10 (tandem stand eyes closed)^15^.

As for floor and ceiling effects, on our sample of older people living in long-term care institutions/nursing homes, we identified low floor and low ceiling effects. Our findings are in accordance with previous studies that reported floor effects ranging from 0 to 2.5% and ceiling effects ranging from 0 to 12.5% and are particularly very close to the results of the study on the Australian sample of older people under acute hospital care, where 1% floor effect and 3.8% ceiling effect were published.

The Cronbach’s alpha coefficient is an indicator of the internal consistency of the scale, and values between 0.7 and 0.95 are considered to be a higher consistency index^31^. In our study, the Cronbach’s alpha was 0.906, which is as good as the results of the Brazilian study (α = 0.9), but a little higher than the values published in the German study (α = 0.87)^21,29^.

The inter-rater reliability in this study was similar to those reported in previous studies, where ICCs ranged from 0.84 to 0.90.

In our study, the MDC_90_ for the DEMMI was 6.803 points, which is less than in the Australian and German studies (where it was 9.51 and 8.8 points, respectively) but higher than in the Brazilian study (where it was 1.83 points)^15,21,29^. However, our result is very close to those of Dutch and Turkish studies (where it was 6.7 and 7.33 points)^24,28^.

The clinical utility (i.e., there were no items to be necessarily excluded) and the clinimetric characteristics (the mutual reinforcement of psychometric indicators, i.e., the synergy between them, and thus the use of Rasch analysis/structural equation modeling) support the identification of both a psychometrically reliable as well as robust and a clinically useful HU-DEMMI scale.

## Limitations

Although our study is robust, it has limitations. First, the generalizability of our study is limited by the fact that our sample was limited to institutions in the capital, which raises questions about the representativeness of the sample. Future studies sampling patients from nursing homes in different parts of the country may increase the generalizability of our findings. Second, the assessors for both the inter- and intra-rater reliability studies were exclusively physiotherapists with only five and seven years of experience in geriatric physiotherapy, no other staff (health professionals) were involved. Therefore, the reliability results are not necessarily representative of all Hungarian physiotherapists and cannot be generalized to other health professionals. Reliability assessment should therefore be repeated with physiotherapists and nurses with different work experience. Finally, the cross-sectional design of the study is also a limitation, so future studies may need to establish the (longitudinal) test-retest reliability and longitudinal stability of the HU-DEMMI with longitudinal study design.

## Conclusion

In our study, we successfully developed the Hungarian version of the DEMMI and investigated its psychometric properties among older people living in long-term care facilities.

The DEMMI is a valid and reliable measure of mobility, one of the most important indicators of independent living. Institutionalized older people are particularly vulnerable to a dramatic decline in mobility and consequent decline in functional capacity. Maintaining their functional capacity and delaying decline requires effective intervention. To effectively delay the mobility decline, valid and reliable determinations of mobility levels need to be ensured. This can help the therapist to engage older adults in personalized movement therapy tailored to their individual needs.

The valid and reliable Hungarian version of the DEMMI scale would also allow for future comparisons of research results from Hungary with those from other countries. Furthermore, the HU-DEMMI would give Hungarian researchers the opportunity to participate in international, multicenter research.

## Electronic supplementary material

Below is the link to the electronic supplementary material.

Supplementary Material 1

Supplementary Material 2

## Data Availability

Sequence data that support the findings of this study have been deposited in the OSF repository, https://osf.io/q349r/?view_only=c933f103fab6450381eff3b636a1c72a DOI: 10.17605/OSF.IO/Q349R.

## Abbreviations

30sSTST: 30 s Sit to Stand Test
ADL: activities of daily living
BI: Barthel Index
BMI: body mass index
CCI: Charlson Comorbidity Index
CFI: comparative fit index
DEMMI: de Morton Mobility Index
DIF: Differential Item Functioning
FAC: Functional Ambulation Category
FES-I: Falls Efficacy Scale - International
HU-DEMMI: Hungarian version of the DEMMI scale
ICC: intraclass correlation coefficients
IQR: interquartile range
LoA: limits of agreement
LOS: Length of Stay (in the institution)
MDC: minimally detectable change
MMSE: Mini-Mental State Examination
RMSEA: root mean square error of approximation
RO: research objectives
RQ: research questions
SD: standard deviations
SEM: standard error of measurement
TLI: Tucker–Lewis Index
TUG: Timed Up and Go
WLSMV: Weighted Least Squares Mean and Variance
