# Supplementary material for: Validation of the de Morton Mobility Index for measuring mobility related activities in Hungarian institutionalized older adults
Source: Sci Rep. 2025 Jul 23;15:26840. doi: 10.1038/s41598-025-09453-6 (PMC12287279; doi:10.1038/s41598-025-09453-6)
Supplement: Supplementary file 1 — Supplementary Material 1 [file 41598_2025_9453_MOESM1_ESM.doc]

Based on our research objectives, we sought to answer the following research questions (RQ).

Research question related to the research objective 1:

RQ1: Which equivalent terms are required to be chosen during the process of linguistic adaptation to ensure cross-cultural validity?

Research questions related to the research objective 2:

RQ2a: Does the HU-DEMMI show acceptable construct validity, based on an association between the DEMMI and another mobility-related measures?

RQ2b: Does the HU-DEMMI show acceptable construct validity, based on its correlation with non-mobility measures?

RQ2c: Does the HU-DEMMI show acceptable construct validity, based on differences in known groups?

RQ2d: How well does the original unidimensional DEMMI fit our observed data?

RQ2e: Is the requirement of unidimensionality met or somewhat compromised, or if multidimensionality is likely, does a three-dimensional or a five-dimensional model fit the data better than the unidimensional model?

Subgroups were created (i.e., the items of the scale were grouped into subsets, thus defining separate dimensions) based on two possible criteria. These were defined on the basis of the DEMMI item groups on the one hand, and their mobility level on the other.

Accordingly, in our five-dimensional model, dimensions were defined according to DEMMI item groups (i.e., A-B-C-D-E).

In our three-dimensional model, dimension 1 comprises the set of items in which the items are not mobility activities that change location, but mobility activities that change position. These items are mostly activities defined as low-level mobility, i.e., bed mobility and chair mobility (A-B) [1, 2].

Dimension 2 includes the item groups of walking on flat ground and, as a direct prerequisite for walking on flat ground, static standing. (C-D) [3].

Dimension 3 includes a group of challenging tasks such as items “Pick up the pen from the floor”, “Walks four steps backwards”, and “Jump”.

As for the task “Pick up the pen from the floor”, it is a typical motor-cognitive dual-task situation; a fine-motor-behavioral cognitive task as a secondary manipulation task (reaching forward to grasp a pen with a precision grip) is performed in parallel with a primary static balance task (maintaining posture while leaning forward) [4]. During these dual-task situations, the attention available should be divided between the primary and secondary tasks [5]. Dividing attention becomes increasingly difficult as we age, so maintaining balance in dual-task situations is a major challenge for an older person [5, 6].

As for the “Walk four steps backwards” task, this mobility activity can also be considered a challenge. From a neural control perspective, walking forwards and walking backwards are different motor behaviors with different kinematics and kinetics as well [7]. Walking backward is done in a different visual field than walking forward. When walking backwards, there is no visual cue of the path. At the same time, walking forwards is more automatic because we have adapted to it. This may explain why walking backwards requires greater activation of brain areas associated with visual-spatial processing and sensorimotor control [8]. Furthermore, the spatial gait parameters are larger and the temporal gait parameters are smaller than those of the forward gait parameters [9]. These facts are the reason why walking backwards requires more cognitive effort and postural control than walking forwards [10].

With regards to the task “Jump”, it requires a high degree of coordination from various muscle groups. This mobility activity consists of a downward movement by flexing the knees and hips (with eccentric muscle contraction), immediately followed by the extension of the knees and hips (with a dynamic concentric muscle contraction) [11]. To successfully execute a jump, the skeletal muscles should exert maximum force in the shortest possible time [12]. This muscle function is referred to as muscle power. Muscle power declines faster with age than muscle strength [13].

Research question related to the research objective 3:

RQ3: Is the Hungarian version of the DEMMI applicable and reliable for measuring mobility among older people living in long-term care facilities with different levels of mobility ability?

**References**

1 Wald, H. L. *et al.* The Case for Mobility Assessment in Hospitalized Older Adults: American Geriatrics Society White Paper Executive Summary. *Journal of the American Geriatrics Society* **67**, 11-16, doi:<https://doi.org/10.1111/jgs.15595> (2019).

2 Brown, C. J. & Flood, K. L. Mobility Limitation in the Older Patient: A Clinical Review. *JAMA* **310**, 1168-1177, doi:10.1001/jama.2013.276566 (2013).

3 Hedman, L. D. *et al.* Locomotor Requirements for Bipedal Locomotion: A Delphi Survey. *Physical Therapy* **94**, 52-67, doi:10.2522/ptj.20120514 (2014).

4 Bayot, M. *et al.* The interaction between cognition and motor control: A theoretical framework for dual-task interference effects on posture, gait initiation, gait and turning. *Neurophysiologie Clinique* **48**, 361-375, doi:<https://doi.org/10.1016/j.neucli.2018.10.003> (2018).

5 Woollacott, M. & Shumway-Cook, A. Attention and the control of posture and gait: a review of an emerging area of research. *Gait & Posture* **16**, 1-14, doi:<https://doi.org/10.1016/S0966-6362(01)00156-4> (2002).

6 Verhaeghen, P. & Cerella, J. Aging, executive control, and attention: a review of meta-analyses. *Neuroscience & Biobehavioral Reviews* **26**, 849-857, doi:<https://doi.org/10.1016/S0149-7634(02)00071-4> (2002).

7 Moon, Y. & Bae, Y. The effect of backward walking observational training on gait parameters and balance in chronic stroke: randomized controlled study. *European Journal of Physical and Rehabilitation Medicine* **58**, 9-15, doi:10.23736/s1973-9087.21.06869-6 (2022).

8 Ogawa, T., Obata, H., Yokoyama, H., Kawashima, N. & Nakazawa, K. Different functional networks underlying human walking with pulling force fields acting in forward or backward directions. *Scientific Reports* **13**, 1909, doi:10.1038/s41598-023-29231-6 (2023).

9 Balasukumaran, T., Olivier, B. & Ntsiea, M. V. The effectiveness of backward walking as a treatment for people with gait impairments: a systematic review and meta-analysis. *Clinical Rehabilitation* **33**, 171-182, doi:10.1177/0269215518801430 (2018).

10 Callisaya, M. L., Blizzard, L., McGinley, J. L., Schmidt, M. D. & Srikanth, V. K. Sensorimotor Factors Affecting Gait Variability in Older People—A Population-Based Study. *The Journals of Gerontology: Series A* **65A**, 386-392, doi:10.1093/gerona/glp184 (2010).

11 Rava, A. *et al.* Body Composition, Neuromuscular Performance, and Mobility: Comparison Between Regularly Exercising and Inactive Older Women. *Journal of Aging and Physical Activity* **25**, 58-64, doi:10.1123/japa.2016-0019 (2017).

12 Moran, J., Ramirez-Campillo, R. & Granacher, U. Effects of Jumping Exercise on Muscular Power in Older Adults: A Meta-Analysis. *Sports Medicine* **48**, 2843-2857, doi:10.1007/s40279-018-1002-5 (2018).

13 Reid, K. F. *et al.* Longitudinal decline of lower extremity muscle power in healthy and mobility-limited older adults: influence of muscle mass, strength, composition, neuromuscular activation and single fiber contractile properties. *European Journal of Applied Physiology* **114**, 29-39, doi:10.1007/s00421-013-2728-2 (2014).
