# Supplementary material for: Validation of the de Morton Mobility Index for measuring mobility related activities in Hungarian institutionalized older adults
Source: Sci Rep. 2025 Jul 23;15:26840. doi: 10.1038/s41598-025-09453-6 (PMC12287279; doi:10.1038/s41598-025-09453-6)
Supplement: Supplementary file 2 — Supplementary Material 2 [file 41598_2025_9453_MOESM2_ESM.pdf]

de MORTON MOBILITÁSI INDEX (DEMMI)

Kérem, olvassa el figyelmesen a 2. oldalon lévő tájékoztatót!

	0	1	2
**A.) Ágy**			
1. Medenceemelés	☐ nem képes	☐ képes	
2. Oldalra fordulás	☐ nem képes	☐ képes	
3. Fekvésből felülés	☐ nem képes	☐ min. segítséggel<br>☐ felügyelettel	☐ önállóan
**B.) Szék**			
4. Széken ülés támaszték nélkül	☐ nem képes	☐ 10 mp	
5. Székről, ülésből felállás	☐ nem képes	☐ min. segítséggel<br>☐ felügyelettel	☐ önállóan
6. Ülésből felállás a karok használata nélkül	☐ nem képes	☐ képes	
**C.) Statikus egyensúly (járási segédeszköz nélkül)**			
7. Állás támaszték nélkül	☐ nem képes	☐ 10 mp	
8. Állás zárt lábfejekkel	☐ nem képes	☐ 10 mp	
9. Lábujjhegyen állás	☐ nem képes	☐ 10 mp	
10. Tandemállás (tyúklépésben állás) csukott szemmel	☐ nem képes	☐ 10 mp	
**D.) Járás**			
11. Egy bizonyos táv megtétele +/- járási segédeszköz<br>*járási segédeszköz (bekarikázni) semmi/járókeret/bot/egyéb*	☐ nem képes<br>☐5 m	☐ 10 m<br>☐ 20 m	☐ 50 m
12. A járás önállósága	☐ nem képes<br>☐ min. segítséggel<br>☐ felügyelettel	☐ önállóan járási segédeszközzel	☐ önállóan járási segédeszköz nélkül
**E.) Dinamikus egyensúly (járási segédeszköz nélkül)**			
13. Toll felvétele a padlóról	☐ nem képes	☐ képes	
14. 4 lépés hátrafelé	☐ nem képes	☐ képes	
15. Ugrás	☐ nem képes	☐ képes	
**OSZLOPONKÉNTI ÖSSZPONTSZÁM**			

NYERS ÖSSZPONTSZÁM (oszloponkénti összpontszámok összege) /19

DEMMI PONTSZÁM (MDC_{90} = 9 pont; MCID = 10 pont) /100

legkönnyebb

- ülés támaszték nélkül
- medenceemelés
- állás támaszték nélkül
- ülésből felállás
- fordulás
- fekvésből felülés
- állás zárt lábfejekkel
- toll felvétele a padlóról hátrafelé járás
- egy bizonyos táv megtétele
- ülésből felállás karhasználat nélkül
- önálló járás
- ugrás
- lábujjhegyen állás
- tandemállás csukott szemmel

legnehezebb

Nyers-DEMMI átváltási táblázat

**Nyers pontszám**	0	1	2	3	4	5	6	7	8	9	10	11	12	13	14	15	16	17	18	19
**DEMMI pontszám**	0	8	15	20	24	27	30	33	36	39	41	44	48	53	57	62	67	74	85	100

Megjegyzések:

Aláírás: Dátum:

Használati útmutató

Fogalommeghatározások
Minimális segítséggel = "kézzel támogatott" fizikai, de minimális segítség, elsősorban a mozgás irányítására.
Felügyelettel = egy másik személy felügyeli a tevékenységet anélkül, hogy kézzel segítséget nyújtana. Szóbeli irányítást adhat.
Önállóan = egy másik személy jelenléte nem szükséges a biztonságos mozgáshoz.

A.) Ágy

1. A személy hanyatt fekszik, és arra kérjük, hogy hajlítsa be a térdét, és emelje fel a fenekét az ágyról.
2. A személy hanyatt fekszik, és arra kérjük, hogy külső segítség nélkül forduljon az egyik oldalára.
3. A személy hanyatt fekszik, és arra kérjük, hogy üljön fel az ágy szélére.

B.) Szék

4. A személyt arra kérjük, hogy 10 másodpercig tartsa meg az egyensúlyát a széken ülve, anélkül, hogy a karfára támaszkodna, görnyedne vagy meginogna. A hát és a karok nem érintkezhetnek a székkel. A térdek és a lábak összezárva, és a talpak a padlón vannak.
5. A személyt arra kérjük, hogy a szék karfáit használva emelkedjen fel ülésből állásba.
6. A személyt arra kérjük, hogy álljon fel a mellkasán keresztbe tett karral.

C.) Statikus egyensúly

7. A személyt megkérjük, hogy álljon 10 másodpercig külső támasz nélkül.
8. A személyt megkérjük, hogy álljon 10 másodpercig összezárt lábfejekkel.
9. A személyt megkérjük, hogy álljon 10 másodpercig lábujjhegyen.
10. A személyt megkérjük, hogy helyezze az egyik lábfej sarkát közvetlenül a másik elé és ezt 10 másodpercig tartsa csukott szemmel.

D.) Járás

11. A személyeket arra kérjük, hogy az épp használt járási segédeszközükkel menjenek, ameddig pihenő nélkül képesek. A teszt véget ér, ha a személy megáll pihenni. A személy a számára legmegfelelőbb járási segédeszközt használja. Ha két járási segédeszköz közül bármelyiket lehet használni, akkor azt a segédeszközt kell használni, amelyik a személy számára a legnagyobb fokú önállóságot biztosítja. A teszt véget ér, ha a személy eléri az 50 métert.
12. Az önállóságot a személy 11. pont feladata során mért maximális gyaloglástávolsága alatt (de legfeljebb 50 méterig) vizsgáljuk.

E.) Dinamikus egyensúly

13. A tollat 5 cm-re helyezzük az álló személy lába elé. A személyt megkérjük, hogy emelje fel a tollat a padlóról.
14. Sétáljon hátrafelé 4 lépést. A személy mindvégig őrizze meg egyensúlyát.
15. A személy képes ugrani. Mindkét lába elemelkedik a talajtól. A személy mindvégig őrizze meg egyensúlyát.

A DEMMI ALKALMAZÁSÁNAK PROTOKOLLJA

1. A vizsgálatot a **beteg ágya mellett** kell elvégezni.
2. A vizsgálatot akkor kell elvégezni, amikor a személy a számára **előírt gyógyszerek megfelelő hatása alatt** van, pl. legalább fél órával a fájdalomcsillapító vagy Parkinson-kór elleni gyógyszeres kezelés után.
3. A vizsgálatot az A-E. szakaszokban **leírt sorrendben** kell elvégezni: ágyban történő helyzetváltoztatás (transzfer), székről történő helyzetváltoztatás (transzfer), statikus egyensúly, járás és dinamikus egyensúly.
4. Minden egyes feladatot **el kell magyarázni**, és ha szükséges, **be kell mutatni** a személynek.
5. A feladatoknál **pipával kell jelezni** a feladat sikerességét vagy sikertelenségét. Ha a feladat tesztelése elmarad, annak okait fel kell jegyezni.
6. **A feladatokat nem szabad elvégezni, ha akár a vizsgálatot végző, akár a feladatot végrehajtó személy nem szívesen próbálkozik a feladattal.**
7. A személyeket az **első próbálkozás** alapján kell pontozni.
8. Ha egy feladat **nem alkalmazható megfelelően a személy egészségi állapota miatt**, akkor a feladatot nem szabad tesztelni, és ennek **okát fel kell jegyezni**.
9. A személyeket lehet bátorítani, de **nem szabad visszajelzést adni** a teljesítményükről.
10. **Három eszközre van szükség**: 45 cm-es ülésmagasságú **szék** karfával, egy kórházi **ágy** vagy kezelőágy és egy **toll**.
11. A vizsgálatot végző személy a vizsgálat során kezelheti a **személy orvosi segédeszközei**t. (pl. hordozható oxigén, infúzió, drén stb.), kivéve, ha a személynek minimális segítségre van szüksége a feladat elvégzéséhez, és ekkor egy második személyre van szükség az orvosi eszközök kezeléséhez.
12. Azoknak a személyeknek, akiknek **légszomj miatt** minden egyes feladat után **pihenés**re van szükségük, 10 percet kell pihenniük, a **tesztelés felénél**, azaz a széktranszferek elvégzése után 10 perc pihenőt kell biztosítani.
13. Azoknak a személyeknek, **akik kevéssé mobilisak**, és emelőt igényelnek az ágyba/az ágyból való átjutáshoz, vagy a székre történő átjutáshoz, a székre történő átjutás szakasza az ágyra történő átjutás előtt is elvégezhető (**A és B szakasz felcserélhető**).
14. **Ágyban történő helyzetváltoztatás (transzfer)**: az ágy magasságának az adott személy számára megfelelőnek kell lennie. Szabványosított kórházi ágyat vagy kezelőágyat kell használni a teszteléshez. A személy nem használhat külső segítséget, például kapaszkodót, ágyrácsot, az ágy szélét vagy ágyrudat. További párnákat lehet biztosítani azon személyek számára, akik nem tudnak hanyatt feküdni.
15. **Székről történő helyzetváltoztatás (transzfer)**: Egy szabványos 45 cm-es ülésmagasság szükséges. Stabil, karfás széket kell használni.
16. **Egyensúly**: Az egyensúly vizsgálatakor nem lehet cipőt viselni. A személy nem használhat külső támaszt az egyensúlyi feladatok sikeres elvégzéséhez. Az ülőegyensúly vizsgálatakor sem a karfa, sem a szék háttámlája nem használható külső támaszként. Az álló egyensúlyi teszteket úgy kell elvégezni, hogy a személy az egyik oldalon egy megemelt ágy, a másik oldalon a vizsgálatot végző személy között áll. Ha a személy bizonytalanságot mutat vagy jelentősen meginog a vizsgálat során, az adott feladat vizsgálatát le kell állítani.
17. **Járás**: A járástesztekhez megfelelő cipőt lehet viselni. Ugyanazt a cipőt kell viselni a vizsgálat megismétlésekor.
18. **Pontozás**: A mellékelt átváltási táblázat segítségével a nyers pontszámot át kell váltani egy DEMMI-PONTSZÁMRA.

Eredeti cím: de Morton Mobility Index (DEMMI). Copyright de Morton, Davidson & Keating 2007.
A DEMMI változtatás nélkül kinyomtatható vagy sokszorosítható (a szerzői jogi megjegyzés megtartásával). Minden egyéb jog fenntartva. Egyéb engedélyekért (beleértve a DEMMI fordítását) forduljon Dr. Natalie de Mortonhoz: natalie.demorton@med.monash.edu.au
Magyar fordítás: de Morton Mobilitási Index (DEMMI).
A DEMMI fejlesztését az Ausztrál Nemzeti Egészségügyi és Orvosi Kutatási Tanács posztgraduális ösztöndíja (Dora Lush Posztgraduális Ösztöndíj, 280632. sz. ösztöndíj) támogatta, amelyet a HCF Egészségügyi és Orvostudományi Kutatási Alapítvány és a Northern Klinikai Kutatóközpont, Northern Health is támogat.
A DEMMI-t a következőképpen kell idézni: de Morton NA, Davidson M, Keating JL. The de Morton Mobility Index (DEMMI): an essential health index for an ageing world. Health and Quality of Life Outcomes 2008, 6:63.
